# Clinical evaluation for morbidity associated with soil-transmitted helminth infection in school-age children on Pemba Island, Tanzania

**DOI:** 10.1371/journal.pntd.0007581

**Published:** 2019-07-15

**Authors:** Isaac I. Bogoch, Benjamin Speich, Nathan C. Lo, Wendelin Moser, David Croll, Said M. Ali, Shaali M. Ame, Jürg Utzinger, Jason R. Andrews, Jennifer Keiser

**Affiliations:** 1 Divisions of General Internal Medicine and Infectious Diseases, Toronto General Hospital, University Health Network, Toronto, Canada; 2 Department of Medicine, University of Toronto, Toronto, Canada; 3 Swiss Tropical and Public Health Institute, Basel, Switzerland; 4 University of Basel, Basel, Switzerland; 5 Basel Institute for Clinical Epidemiology and Biostatistics, Department of Clinical Research, University of Basel and University Hospital Basel, Basel, Switzerland; 6 Department of Medicine, University of California, San Francisco, San Francisco, California, United States of America; 7 Division of Infectious Diseases and Geographic Medicine, Stanford University School of Medicine, Stanford, California, United States of America; 8 Laboratory Division, Public Health Laboratory - Ivo de Carneri, Chake Chake, Tanzania; Weill Cornell Medical College, UNITED STATES

## Abstract

**Background:**

More than 1.5 billion people are infected with soil-transmitted helminths (*Ascaris lumbricoides*, hookworm, *Strongyloides stercoralis*, and *Trichuris trichiura*), causing an estimated global burden in excess of 3 million disability-adjusted life years. However, the relationship between soil-transmitted helminth infection, adverse health consequences, and beneficial effects of deworming are not well understood.

**Methodology:**

We pursued a detailed longitudinal clinical evaluation of school-age children to evaluate morbidity associated with soil-transmitted helminth infection and responses to treatment. This exploratory study was embedded into a randomized controlled trial. Overall, 434 children, aged 7–14 years, underwent a detailed medical history, physical examination, stool microscopy for soil-transmitted helminths, and hemoglobin (Hb) measurement at baseline. Medical history and stool examination were repeated at 3 and 18 weeks posttreatment. Additionally, Hb measurement was performed at the 18-week treatment follow-up. Logistic regression was employed to assess clinical factors associated with soil-transmitted helminth infection at baseline, and longitudinal data analysis to examine change in health outcomes following treatment over time.

**Principal findings:**

All enrolled children were infected with *T*. *trichiura*, and randomized into four different treatment interventions. None of the medical history, physical examination, and laboratory (i.e., Hb) findings were associated with *A*. *lumbricoides*, hookworm, or *S*. *stercoralis* infection at baseline. A composite of physical exam findings for anemia, including pallor of the conjunctiva, nail beds, and palmar creases predicted lower Hb values (-3.8 g/dl, 95% confidence interval (CI): -6.9, -0.6 g/dl). When examining longitudinal trends, we did not find improvements to Hb or face Wong-Baker Likert scale among children with soil-transmitted helminth infection compared to those without infection, although there was a slight trend toward improving Hb values after treating hookworm infection.

**Conclusions/significance:**

Our study demonstrates the challenges of measuring morbidity in the context of soil-transmitted helminth infection and treatment, thus confirming the mainly subtle morbidity effects of infection.

## Author summary

Soil-transmitted helminth infections frequently affect children and impoverished individuals in low-resource settings and are associated with negative health effects. We attempted to measure morbidity in school-age children from Pemba Island, Tanzania who are infected with soil-transmitted helminths but with relatively low risk for other infections known to cause chronic morbidity, such as malaria and schistosomiasis. We used a composite of clinical history, physical examination, and hemoglobin measurements. Our study was embedded into a randomized controlled trial, and by design, all individuals were infected with whipworms (*Trichuris trichiura*). A detailed clinical evaluation for morbidity in school-age children was not associated with an infection of roundworms (*Ascaris lumbricoides*), hookworm, or threadworms (*Strongyloides stercoralis*) but demonstrated the utility of the clinical examination to detect anemia. Indeed, anemia slightly trended toward resolution after deworming. While many metrics for soil-transmitted helminth infection-related morbidity may have been masked by ongoing deworming campaigns, simple physical exam findings for anemia can be useful to highlight individuals that require further evaluation.

## Introduction

Soil-transmitted helminths (*Ascaris lumbricoides*, hookworm, *Strongyloides stercoralis*, and *Trichuris trichiura*) affect more than 1.5 billion people worldwide with the highest prevalence and intensity of infection often observed in school-age children in low- and middle-income countries [1, 2]. Chronic infection with soil-transmitted helminths can lead to anemia, malnutrition, stunted growth, and delayed cognitive development [2]. The current global strategy to control infection in high-burden areas aims to reduce morbidity with preventive chemotherapy in regions where the prevalence of infection is above a pre-specified threshold [3, 4], however, re-infection rates remain high [5, 6]. There is some debate over the role of preventive chemotherapy in reducing morbidity in school-age children, and hence, there is a need for rigorous studies to evaluate the impact of soil-transmitted helminths on morbidity and changes after treatment [7, 8]. It is still generally accepted that preventive chemotherapy is beneficial and that contradictory results may be related to how and when morbidity is measured [9–11] but a better clinical understanding of measurable reductions in soil-transmitted helminth infection-related morbidity in response to therapy is now recognized as a priority research area [12].

Several non-invasive strategies have been evaluated to either diagnose soil-transmitted helminth infection or determine the morbidity associated with infection, including the use of anamnestic questionnaires [13] and ultrasonography [14]. To date, studies assessing the utility of a clinical examination aimed at diagnosis or quantifying morbidity associated with soil-transmitted helminth infection have been somewhat limited in scope. Here, we report on the results of a detailed clinical evaluation of school-age children infected with one or several species of soil-transmitted helminths in rural communities on Pemba Island, Tanzania. We evaluated whether a clinical approach was able to detect morbidity associated with soil-transmitted helminth infection, and whether changes to morbidity are clinically detectable in response to treatment.

## Methods

### Ethics statement

Ethical approval for the study was granted by both the *Zanzibar* Medical Ethical Research Committee (reference no. ZAMREC, 0001) and the Ethics Committee of Northwestern and Central Switzerland (reference no. EKBB #123). Children aged 7–14 years from two schools (Mchangamdogo and Shungi) were invited to enroll in this study. All participants had written informed consent from a parent or legal guardian, while children assented orally. Participation was voluntary and children could withdraw anytime without further obligations.

### Study site and design

The study was integrated into a randomized controlled trial (RCT), evaluating novel treatment strategies for *T*. *trichiura* infection in school-age children on Pemba Island, Tanzania. It was carried out between September 2013 and March 2014. Details of the design of the RCT, baseline characteristics, and infection data of school-age children, and results of the efficacy and safety of the different treatment strategies have been reported elsewhere [15, 16].

Selection for the RCT started before this study, and all children enrolled were, by design, infected with *T*. *trichiura* at baseline. The study consisted of a baseline and two follow-up assessments at 3 and 18 weeks after randomization and initial anthelmintic treatment. The baseline evaluation included a detailed medical history, physical examination, stool microscopy for diagnosis of species-specific helminth infection and intensity, and hemoglobin (Hb) finger prick measurement with a HemoCue device (HemoCue 301 system; Ängelholm, Sweden). Children were randomized and treated with either mebendazole, albendazole plus mebendazole, albendazole plus ivermectin, or albendazole plus oxantel pamoate. The first follow-up was performed 3 weeks after randomization and treatment, and included a detailed medical history and stool microscopy. A second follow-up was conducted 18 weeks after baseline testing, and included a detailed medical history, stool microscopy, and a repeat Hb measurement. Hb measurements were conducted at this time to allow for an appropriate time to measure the potential resolution of anemia, as assessed by Hb level [17]. At the end of our study, after the 18-week follow-up survey, all children, regardless of their infection status, were treated with albendazole (400 mg) as per national guidelines.

### Clinical evaluation

A dedicated school classroom was temporarily converted into a clinic space, where clinical evaluations were conducted, and to facilitate laboratory specimen collection. Trained nurses using a standardized questionnaire conducted the medical history component of the clinical examination, directly in Swahili [18]. The first section of the medical history highlighted past medical history, allergies, medication history, and any illness in the 4 weeks before the survey that were severe enough for a child to stay home from school. The medical history then focused on generalized and focal issues, including active symptoms of fever, chills, cough, headache, vertigo, abdominal cramps, fatigue, nausea, vomiting, diarrhea, hematochezia, hematuria, constipation, anorexia, pruritis, ankle edema, dyspnea, and difficulty concentrating. If there were active symptoms present, children were asked to elaborate on the severity and duration of such symptoms. Lastly, children were asked to point to a Wong-Baker Likert scale of six faces [19, 20], that best described their overall health and wellbeing over the past 4 weeks.

Two experienced physicians performed a detailed physical examination. This focused on general characteristics (height, weight, heart rate, respiratory rate, and presence of jaundice), and appearance of malnourishment (temporalis muscle wasting, loss of subcutaneous fat at the deltoid, triceps, interosseous hand muscles, and quadriceps muscle groups [21], and conjunctival evidence of vitamin A deficiency). Children were then evaluated for the presence of abdominal pain with light and deep palpation in four quadrants, the presence of ascites (inspection, pitting ankle edema, flank dullness, and fluid wave [22]), splenomegaly (inspection, Castell’s sign, and palpation [23]), hepatomegaly (inspection, palpation, and hook test [24]), pulmonary hypertension (cyanosis, prominent “a” or “cv” waves on jugulovenous pressure, Kussmaul’s sign, palpable P2 on precordial exam, a murmur of tricuspid regurgitation, or the presence of right sided S3 or S4 heart sound [25]) in addition to a general cardiac and pulmonary exam via auscultation. Auscultation was performed with double lumen stethoscopes (Littmann, 3M; St. Paul, MN, United States of America). Lastly, signs of anemia were recorded and included evaluation for pallor of the conjunctiva, nail beds, and palmar creases, in addition to evidence of koilonychia, angular chelitis, or glossitis [26, 27]. Data were collected on paper forms and then entered in duplicate into Microsoft Excel files (Microsoft; Redmond, WA, United States of America).

### Laboratory procedures

Children provided stool samples on two consecutive days for baseline, first follow-up at 3 weeks after randomization and treatment, and second follow-up at 18 weeks posttreatment. Samples were transferred to the Public Health Laboratory—Ivo de Carneri for processing on the day of collection. Duplicate Kato-Katz thick smears were made from each stool sample on both days, using a 41.7 mg template [28]. Kato-Katz thick smears were examined under a microscope by one of six experienced laboratory technicians for the presence and quantification of eggs from *A*. *lumbricoides*, hookworm, and *T*. *trichiura*. All stool samples were processed the same day and slides read under a microscope within one hour of preparation to account for rapid hookworm egg disintegration [29]. For quality control, 10% of slides were randomly selected and re-read by a third, senior microscopist and the results were discussed until consensus was reached. Additionally, stool samples were examined for *S*. *stercoralis* infection via Koga-agar plate and Baermann techniques [30]. The presence of larvae by one or both of these techniques constituted a positive test.

### Statistical analysis

We included children who had written informed consent from their parents/legal guardians, assented orally, and had complete data records for their parasitologic (quadruplicate Kato-Katz thick smear readings for all three time points), and clinical history and physical exam outcomes. We reported helminth-specific prevalence and arithmetic mean and their corresponding 95% confidence intervals (CIs) for infection intensity (as expressed by eggs per 1 g of stool; EPG) at the three study time points. Using data on clinical history and physical exam, we assessed clinical factors associated with each soil-transmitted helminth infection. We selected clinical variables that were pre-specified based upon clinical expertise and excluded rarely reported outcomes (<2% of study population). We used six pre-defined aspects of the history based on clinical opinion (abdominal pain, blood in stool, diarrhea, headache, recent sick days, and Wong-Baker Likert scale), three physical exam features (a composite of anemia findings and malnutrition findings, and height), and one laboratory parameter (Hb) to determine clinical associations for infection with *A*. *lumbricoides*, hookworm, or *S*. *stercoralis*. Clinical variables associated with *T*. *trichiura* could not be conducted as, by design of the RCT, only children with an infection of *T*. *trichiura* at baseline were included. Examination for abdominal pain on physical examination was not used as all evaluations were benign. There were a total of 30 statistical comparisons (10 history, physical exam, and laboratory factors—including composite physical exam findings, with three binary parasitologic infection outcomes with each helminth species) in univariate analyses with logistic regression where infection with each soil-transmitted helminth was the dependent variable. We applied a Bonferroni correction to adjust for multiple comparisons for interpretation of our conclusions.

We investigated the longitudinal change in pre-defined health outcomes (Hb and face Wong-Baker Likert scale) following treatment using the two follow-up time points. For Hb, where only two measurements were available (baseline and 18-week posttreatement follow-up), we used an unpaired *t*-test to examine change in Hb in individuals with and without each helminth infection at baseline. We also examined the relation between change in Hb and change in infection intensity, as expressed in EPG with the Spearman correlation coefficient given overdispersion of helminth egg output values. For Wong-Baker Likert scale, where three measurements were available, we used a generalized estimating equation with exchangeable correlation structure and robust standard errors to examine the longitudinal relationship between changes in Wong-Baker Likert scale (dependent variable) over time and baseline helminth infection status (independent variable). Additionally, Wong-Baker Likert scores and Hb levels for each treatment arm were evaluated individually with means and 95% CIs. Data were recorded in a Microsoft Excel spreadsheet, and statistical analysis was performed with R 3.1.1 (R Foundation for Statistical Computing; Vienna, Austria). This study was embedded into a RCT evaluating treatments for *T*. *trichiuris* infection, and the sample size was chosen to power this RCT in which our study was nested. We did not conduct a separate sample size analyses and therefore all analyses used this convenience sample and are of exploratory nature rather than conclusive.

## Results

Overall, 434 of 594 eligible children completed all baseline and follow-up tests and were included in the final analysis. The mean age of our child cohort was 8.3 years (range 7–14 years) and there were 210 (48.4%) females. Table 1 summarizes children’s infection status, stratified by soil-transmitted helminth species. All 434 children were positive for *T*. *trichiura* at baseline, as this was required for inclusion into the RCT. Across all treatment arms, the prevalence of *T*. *trichiura* was 70.3% and 71.7% at the 3-week and 18-week follow-up visits, respectively. Overall, *A*. *lumbricoides* prevalence was 42.2%, 1.6%, and 27.4% at baseline, 3-week, and 18-week posttreatment follow-up visits, respectively. The respective hookworm prevalence was 42.6%, 30.2%, and 30.6%.

**Table 1:** Soil-transmitted helminth infection among 434 school-age children on Pemba Island, Tanzania in late 2013/early 2014 at baseline and 3-week and 18-week treatment follow-up surveys.

Characteristics of soil-transmitted helminth infection	Baseline	3-week treatment follow-up survey	18-week treatment follow-up survey
***Ascaris lumbricoides***			
Prevalence (%)	42.2	1.6	27.4
Prevalence, moderate-heavy infection (%)*	18.0	0.5	5.5
EPG (95% CI)†	4,319 (3,184; 5,581)	110 (3; 289)	942 (612; 1,318)
**Hookworm**			
Prevalence (%)	42.6	30.2	30.6
Prevalence, moderate-heavy infection (%)*	1.2	0.2	0.0
EPG (95% CI)†	118 (82; 160)	48 (30; 73)	41 (29; 55)
***Trichuris trichiura***			
Prevalence (%)	100	70.3	71.7
Prevalence, moderate-heavy infection (%)*	30.2	10.1	9.2
EPG (95% CI)†	1,092 (933; 1,278)	500 (376; 650)	396 (286; 538)
***Strongyloides stercoralis***			
Prevalence (%)	2.5	0.2	0.2

CI, confidence interval; EPG, eggs per 1 g of feces, computed as arithmetic mean in entire study population, including those without infection.

* Moderate and heavy infection intensities for soil-transmitted helminths were according to guidelines put forth by the World Health Organization (WHO) [3]; *A*. *lumbricoides*, ≥5,000 EPG; hookworm, ≥2,000 EPG; *T*. *trichiura*, ≥1,000 EPG

^**†**^ EPG standard errors were computed by bootstrap procedure.

There were no history, physical examination, or laboratory (Hb) findings that were associated with infection for *A*. *lumbricoides*, hookworm, or *S*. *stercoralis* infection at baseline (Table 2). The composite physical exam finding for anemia, including pallor of the conjunctiva, nail beds, and palmar creases, were associated with lower absolute Hb values (-3.8 g/dl, 95% CI: -6.9, -0.6 g/dl).

**Table 2:** Relationship between clinical history and physical exam with soil-transmitted helminth infection among 434 school-age children on Pemba Island, Tanzania in a baseline survey conducted in late 2013*.

Description	*Ascaris lumbricoides*		Hookworm		*Strongyloides stercoralis*	
	OR (95% CI)	*P*	OR (95% CI)	*P*	OR (95% CI)	*P*
***History***						
**Abdominal pain**	0.22 (0.03, 0.82)	0.04	2.50 (0.85, 8.24)	0.11	--	--
**Blood in the stool**	2.46 (0.73, 9.50)	0.16	1.13 (0.32, 3.79)	0.85	--	--
**Diarrhea**	--	--	0.22 (0.01, 1.30)	0.16	--	--
**Headache**	0.68 (0.21, 1.94)	0.48	0.89 (0.29, 2.52)	0.83	--	--
**Recent sick days**	1.09 (0.53, 2.20)	0.81	1.57 (0.78, 3.20)	0.21	--	--
**Wong-Baker Likert scale**	1.02 (0.90, 1.16)	0.74	0.94 (0.83, 1.07)	0.35	1.02 (0.68, 1.51)	0.91
***Physical exam***						
**Signs of anemia**	0.80 (0.43, 1.48)	0.49	1.40 (0.76, 2.56)	0.27	--	--
**Height (cm)**	1.02 (0.99, 1.05)	0.16	1.00 (0.98, 1.02)	0.90	0.99 (0.96, 1.07)	0.78
**Signs of malnutrition**	1.25 (0.79, 1.97)	0.34	0.93 (0.59, 1.46)	0.75	1.31 (0.28, 4.64)	0.69
***Laboratory***						
**Hemoglobin (g/dl)**	0.99 (0.98, 1.01)	0.46	0.98 (0.96, 1.00)	0.05	0.98 (0.93, 1.04)	0.53

CI, confidence interval; OR, odds ratio

*Univariate logistic regressions are presented. Variables without estimates did not include study participants with infections.

Table 3 outlines the relationship between soil-transmitted helminth infection intensity with the two key outcomes of faces on Wong-Baker Likert scale and Hb values, both pre- and posttreatment. There was no relation in the face Wong-Baker Likert scale for infected *versus* non-infected individuals, at baseline for *A*. *lumbricoides*, hookworm, or *S*. *stercoralis*, or in response to anthelmintic treatment. Similarly, there was no correlation for infection intensity and the Wong-Baker Likert scale for each helminth infection at baseline or in response to treatment.

**Table 3:** Longitudinal relationship of anemia (indirectly assessed by hemoglobin level) and Wong-Baker Likert scale quality of life measure after anthelmintic treatment in a study conducted among school-age children on Pemba Island, Tanzania in late 2013/early 2014*†.

	*Ascaris lumbricoides*			Hookworm			*Strongyloides stercoralis*		
	Not infected	Infected	*P*	Not infected	Infected	*P*	Not infected	Infected	*P*
Hb **change after treatment (g/dl)^i^**	+0.01	+0.11	0.19	+0.04	+0.07	0.71	+0.05	+0.04	0.91
	Spearman correlation		*P*	Spearman correlation		*P*	Spearman correlation		*P*
Hb **change correlation with infection intensity change^ii^**	-0.07		0.17	-0.09		0.07			--
	Coefficient		*P*	Coefficient		*P*	Coefficient		*P*
**Wong-Baker Likert scale change, infected *versus* uninfected (interaction)^iii^**	-0.08		0.37	0.05		0.58	-0.10		0.61

Hb, hemoglobin

* Note: *Trichuris trichiura* was not included in the analysis since only children with *T*. *trichiura* infection at baseline were included in the trial.

^†^ Statistical testing for outcomes used: (i) unpaired *t*-test; (ii) Spearman correlation coefficient; and (iii) generalized estimating equation.

We measured a borderline association between being infected with hookworm and lower Hb at baseline (-0.2 g/dl Hb, p = 0.05), although the association did not meet significance given correction for multiple comparisons in the analysis. Furthermore, we found a borderline relation between change in Hb after treatment and change in hookworm infection intensity (rho = -0.09, p = 0.07), meaning that reduction in infection intensity after treatment correlated with increases in Hb values. This relation was not present when measuring hookworm infection as a binary variable. We did not find a relation between Hb and infection status for *A*. *lumbricoides* or *S*. *stercoralis*. Additionally, Hb levels and Wong-Baker Likert scores were comparable between the four treatment arms (Table 4).

**Table 4:** Hemoglobin levels and Wong-Baker Likert scale scores stratified by treatment arms in a study conducted among school-age children on Pemba Island, Tanzania in late 2013/early 2014.

	Albendazole plus ivermectin	Albendazole plus mebendazole	Albendazole plus oxantel pamoate	Mebendazole	Overall
**Baseline**					
Sample size (available data)	108	107	110	110	435
Mean Hb in g/dl (95% CI)	12.4 (12.3–12.6)	12.6 (12.4–12.8)	12.3 (12.1–12.5)	12.6 (12.4–12.8)	12.5 (12.4–12.6)
Mean Wong-Baker Likert score (95% CI)	2.93 (2.65–3.20)	2.82 (2.52–3.12)	2.83 (2.56–3.09)	2.85 (2.55–3.16)	2.86 (2.71–3.00)
**1^st^ follow-up at 3 weeks posttreatment**					
Sample size (available data)	108	105	108	108	429
Mean Wong-Baker Likert score (95% CI)*	3.19 (2.90–3.47)	3.17 (2.89–3.45)	3.19 (2.89–3.50)	3.16 (2.88–3.44)	3.18 (3.04–3.32)
**2^nd^ follow-up at 18 weeks posttreatment**					
Sample size (available data)	105	104	104	107	420
Mean Hb in g/dl (95% CI)	12.6 (12.3–12.8)	12.7 (12.5–12.9)	12.4 (12.2–12.6)	12.5 (12.3–12.8)	12.6 (12.5–12.7)
Mean Wong-Baker Likert score (95% CI)*	2.72 (2.48–2.96)	2.50 (2.26–2.74)	2.39 (2.13–2.66)	2.45 (2.20–2.69)	2.51 (2.39–2.64)

CI, confidence interval; Hb, hemoglobin

## Discussion

This study pursued a detailed clinical examination to determine whether soil-transmitted helminth infection cause measurable morbidity in school-age children in a highly endemic area on Pemba Island, Tanzania, and to monitor potential change in morbidity following anthelmintic treatment. We found a weak association between morbidity and soil-transmitted helminth infection, as there was a slight trend toward improving Hb values following the treatment of hookworm infection. This finding corroborates with results from an RCT conducted in 6- to 14-year-old children in Côte d’Ivoire, where anthelmintic treatment slightly improved Hb values [31].

Several non-invasive and non-laboratory-based methods such as questionnaires, have been used to predict and screen for helminth infection, however many of these studies focused specifically on schistosomiasis. Questionnaires have been employed to rapidly screen large cohorts, such as school-age children in endemic settings who are at greatest risk for infection, and demonstrate that simple metrics, such as reported blood in the urine or stool, is associated with *Schistosoma haematobium* and *S*. *mansoni* infection, respectively [13, 32, 33]. Similarly, simple and rapid clinical tests of morbidity, such as detecting blood in urine with inexpensive urine reagent strips, has demonstrated predictive value for *S*. *haematobium* diagnoses in community settings with high [34, 35] and low *S*. *haematobium* prevalence [36], and in clinical settings [37].

Despite the long history evaluating rapid, non-invasive, and non-laboratory-based methods for the diagnosis of schistosomiasis, there is a paucity of data evaluating such tools for soil-transmitted helminth infection [38], even though co-infection and polyparasitism is common [39, 40]. Similarly, there is a lack of data using a detailed medical history, coupled with an evidence-based clinical examination to evaluate for soil-transmitted helminth infection using morbidity markers, as done in this study. The benefits of such a detailed clinical approach is that it involves well-trained personnel rather than relying on limited laboratory capacity or expensive equipment [41]. The claim is that an evidence-based clinical approach could detect subtle morbidity (e.g., with questions of general well-being), in addition to evaluating for targeted organ system dysfunction from soil-transmitted helminth infection [42]. In this study, the six-point Wong-Baker Likert scale for general pain and discomfort had no predictive value for soil-transmitted helminth infection or anemia. Although these scales have been validated in children in different socio-cultural settings and are widely used [20, 43, 44], they were not useful in the current setting to distinguish potential reductions in helminth-related morbidity at two time points (3 and 18 weeks) after anthelmintic treatment. However, a detailed clinical approach using a composite of validated physical examination findings was effective at detecting anemia [26, 27]. Such physical exam findings have previously been used to detect anemia in school-age children in African settings [45], and are a simple, rapid, and helpful tool that may prompt public health providers or clinicians to perform a more detailed evaluation for anemia with phlebotomy. While the etiology of anemia in children residing in low- and middle-income countries is multifactorial [46, 47], hookworm infection is known to be a common cause of iron-deficiency anemia [48] and improves with treatment [31, 49]. We detected only a very modest improvement in anemia after anthelmintic therapy at 18 weeks after treatment, and these modest results are likely due to the relatively low prevalence of moderate and heavy hookworm infection intensity in this cohort, and that we did not provide iron supplementation.

Metrics of morbidity associated with soil-transmitted helminth infection have been detected by a variety of methods, including measuring several anthropometric features such as height, weight, and head circumference in children [50, 51], and physical fitness of both school-age children and adults [52, 53]. Other studies have not demonstrated such associations between soil-transmitted helminth infection and morbidity [54]. Although preventative chemotherapy campaigns aimed at school-age children in high-prevalence settings have shown mixed results in the mitigation of morbidity [8], it is generally believed that periodic deworming is beneficial in these settings, and that some null results are due to a paucity of sensitive morbidity metrics, shorter-term studies, and the dilution of severe morbidity with ongoing preventative chemotherapy efforts [10]. The conflicting results in many of these studies are also reflective of the many challenges to accurately measure soil-transmitted helminth infection-related morbidity in real world settings. This study was conducted in schools with prior soil-transmitted helminthiasis treatment which could have biased our study findings toward demonstrating little benefit of treatment on metrics that measure morbidity.

The current study setting is well suited to evaluate morbidity from soil-transmitted helminthiasis, since other parasitic diseases, such as urogenital schistosomiasis, lymphatic filariasis, and malaria are nearly eliminated [55–57]. Although soil-transmitted helminth re-infection rates in endemic settings as Pemba Island are generally high, there were only relatively few children with moderate-to-heavy hookworm and *A*. *lumbricoides* infections in the two participating schools, possibly due to frequent treatment within preventive chemotherapy campaigns both for soil-transmitted helminthiasis and lymphatic filariasis, which likely dilutes measurements of morbidity. Other weaknesses of this study include that, by design, all children at baseline were infected with *T*. *trichiura*, so morbidity from this infection could not be evaluated. This study was integrated into an RCT with a sample size chosen to power that trial, hence our analyses are exploratory in nature. Additionally, only those children who attended school were included in the study, thereby potentially eliminating children who were too unwell to attend school. Finally, the 18 week follow-up period may not have been sufficient time to accurately detect changes in morbidity following anthelmintic treatment.

A detailed clinical evaluation for morbidity in school-age children was not associated with *A*. *lumbricoides* or *S*. *stercoralis* infection but demonstrated the utility of the clinical examination to detect anemia, and anemia slightly trended toward resolution after anthelmintic therapy. While many metrics for soil-transmitted helminth infection-related morbidity may have been masked by ongoing preventive chemotherapy efforts, simple physical exam findings for anemia can be useful to highlight individuals that require further evaluation.

## Supporting information

S1 STROBE Checklist (PDF)
